# Does oil price uncertainty matter in stock market volatility forecasting?

**DOI:** 10.1371/journal.pone.0277319

**Published:** 2022-12-28

**Authors:** Peng Qin, Manying Bai

**Affiliations:** Department of Economics and Management, Beihang University, Beijing, China PR; Sapienza University of Rome, ITALY

## Abstract

We analyze whether oil price uncertainty and U.S. stock uncertainty can simultaneously provide additional information to volatility forecast of six major stock indexes. For model settings, we find not only the uncertainty information of previous day, but that of previous week and month will also provide incremental predictive power for the stock market volatility. Based on that, from in-sample and out-of-sample perspective, the empirical evidences imply separately incorporating oil price uncertainty into the model can significantly improve the stock market volatility forecasting performance, but the improvements vanish after controlling the effects of volatility spillover from U.S. stock market while the effect of U.S. stock uncertainty is nonnegligible and sustainable for stock volatility forecasting. We confirm this finding from average and dynamic perspective. We further proceed the process in longer-horizon volatility forecasting, the evidences cannot overturn our conclusion. This conclusion implies that we should be cautious about the stock volatility predictability based on the oil price uncertainty, which further provide some important implications for researchers, regulators and investors.

## 1. Introduction

Volatility forecasting of stock market is a key topic for investors, risk managers and market regulators. Besides the self-evolvement of stock market volatility (e.g. Today’s stock market volatility is affected by stock market volatility in previous periods), the external information may provide additional value for volatility forecasting (e.g. Perhaps the information of B market can be used to predict the volatility of A stock market). There are two types of external information attract much attention when focus on stock volatility investigation. The first type of external information is derived from U.S. stock market. As U.S. stock market is the world ’ s largest and most important stock market, the information of U.S. market uncertainty tends to diffuse to the participants of other stock markets and further impact that stock market [1]. Accordingly, U.S. stock market uncertainty could be treated as external information to improve stock volatility forecasting performance and some researches pay attention to that. For instance, [2] shows the significant improvement of volatility forecasting of international equity markets when the model incorporating the information of U.S. stock uncertainty. Besides the external information derived from U.S. stock market, the second type of external information is derived from oil market. As oil is one of most important energy sources, oil market uncertainty could impact the economy system and further impact stock market. Some researches study oil-stock nexus and report the significant volatility spillover from oil market to stock market (see [3] for a review). However, little out-of-sample evidences that the information of oil market uncertainty can be utilized to improve the stock volatility forecast performance were shown. Until recently, [4] provide evidence that oil volatility risk exhibits significant in-sample and out-of-sample volatility forecasting power for G7 countries. [5] provide similar idea and they construct the oil price uncertainty via monthly realized volatility based on daily oil price series.

We can see [2, 4] show empirical evidence that U.S. stock market and oil market can separately provide additional useful information for stock market volatility forecasting, while another important and interesting question is whether they can simultaneously work. That is, whether the forecast performance obtained by considering both two types of external information is better than those by considering only one type of external information. The investigation of this question is meaningful. If the answer is yes, we can improve forecast performance of [2, 4] by additionally incorporating the information of oil market/U.S. stock market uncertainty. If the answer is no, that may imply one of the two type external information seems to be redundant for stock volatility forecasting and the investors should be cautious about that. Moreover, some researches try to find some other external indicators which can improve the stock volatility predictions. Besides oil market and U.S. stock market information mentioned above, for example, [6] shows the simple mean and first principal component of international stock markets RV (realized volatility) can significantly improve the volatility forecasting performance of individual stock markets. [7] finds the information of implied volatility index of individual stock market can provide improvement of its own volatility forecasting. Maybe some other effective indicators will be found in future, but an interesting and important problem is whether all (or some) of them can simultaneously work in volatility forecasting? That problem could be handled by our framework but is out of our scope, we focus on two types of external indicators that attract much attention as mentioned before. Specifically, this study tries to investigate whether both oil market uncertainty and U.S. stock market uncertainty can simultaneously provide additional useful information to stock volatility forecasting.

In light of the growing related studies on oil volatility/uncertainty and its effect on financial/stock markets, many methods are employed to investigating the related topics. A typical method is multivariate regression. For example, based on standard multivariate regression, [8] investigates the impacts of oil price uncertainty on the stock returns in different industries. The work by [9] use quantile regression methods to investigate the asymmetric relationship between returns and changes in implied moments (i.e., volatility, skewness and kurtosis) in crude oil market. The aforementioned studies are about return-volatility nexus, turn to the volatility connectedness/spillover between crude oil market and stock/financial market (the topic of this study), there are three commonly used methods. The first one is causality test. For instance, the work by [10] study the volatility spillover, pre- and post-crisis, between oil market and stock markets of Lebanon and Jordan by using the causality-in-variance test proposed by [11]. Due to nonlinearity and structural breaks which indicate the misspecification of linear model, [12] employ nonparametric causality-in-quantile framework to reconsider the impact of realized volatility of oil market on term structural of interest rates of the United States. The second commonly used method is multivariate GARCH models. For example, using the ADCC-GARCH-GJR model, [13] examine the time-varying connectedness between oil market and the stock markets of oil importing and oil exporting countries. A multivariate GARCH model is also employed by [14], who explore how oil market shocks affects the stock market volatility. However, multivariate GARCH models have limitation about the so-called curse of dimensionality. The third popular method so-called spillover index proposed and extended by Diebold and [15–18], which simplify the understanding process and derive the direct and indirect spillover across the variables via variance decomposition within VAR model. By using this spillover index, [19] investigate the volatility connectedness between oil market and stock markets from the perspective of implied volatility indexes. [20] also employ that spillover index of [15] to explore the volatility spillover from global economic policy uncertainty and oil market to the stock markets of oil importers and exporters.

However, the aforementioned methods are commonly-used for In-sample analysis but seem inappropriate for Out-of-sample (i.e., forecasting) analysis due to their intrinsic characteristics or limitations in method. With respect to our topic about volatility forecasting, HAR-RV model is suitable due to its simplicity and effectiveness. The method is cascade structure which can capture the important properties of realized volatility such as long memory and multi-scaling behavior. Moreover, the forecasting process of this method is easy to implement. These merits make HAR model attractive and several studies which focus on volatility forecasting base their investigation on this method [2, 6, 21].

We capture the oil market uncertainty and U.S. stock market uncertainty through CBOE crude oil volatility index (OVX for short) and CBOE volatility index (VIX for short), respectively. OVX and VIX are suitable for our purpose as they contain both historical and future information of oil price uncertainty and U.S. stock uncertainty, respectively. More and more researches show evidences that OVX and VIX are more capable price uncertainty proxies than volatility indicators derived from raw price series [2, 22–26]. Since May 10, 2007, the Chicago Board Options Exchange (CBOE) lunched a new implied volatility index (OVX) in the crude oil market; the index, which is similar with VIX in stock market, is calculated conditional on options on the United States Oil Fund and can measure the market’s expectation of the 30-day volatility of crude oil prices [27]. Moreover, they are constructed by same method which makes them comparable. Note that the underlying sectors of OVX and VIX indicate VIX could be treated as the uncertainty representative of all sectors while OVX just represent the uncertainty of oil sector. This will imply the information carried on by the OVX index seems to be contained also in VIX. However, in our opinion, VIX is more likely to contain information about some rather than all of OVX. Some literatures have shown OVX can provide incremental information beyond VIX. For example, through adjusted GARCH models, [28] show, even controlling VIX, OVX can significantly influence the stock market volatilities of most Middle East and Africa countries. Based on quantile regression, after controlling VIX effects, [25] produce the results that OVX show significant positive impact on Chinese stock market volatility under different market conditions. In the two studies, although the coefficients of OVX are smaller than that of VIX which means the impacts of VIX are stronger than that of OVX, the coefficients of OVX are still significant. But in our study, the empirical results imply OVX seems significantly impact the volatility of stock indexes we study, but the influences vanish after controlling the effects of VIX, whereas the impact of VIX is considerable and consistent. Moreover, the results of In-sample and Out-of-sample show, when focus on the volatility forecasting, the longer lagged effects of OVX and VIX should be incorporated into the model as that will improve the forecasting performance. This study provides not a contradiction but rather a good complement to existing literatures and some important implications could be extracted.

This study is closed to [28] as they try to model the impact of OVX on the daily volatility of Middle eastern and African stock markets. Based on GARCH models and In-sample analysis, they conclude that most of those markets can be significantly influenced by OVX even after considering the effect of VIX. But they used daily data and we use intra-day data, and we focus on the six major stock indexes which are not belong to Middle east and Africa. Due to data limitation, we are unable to find the high-frequency data that corresponding to their sample. Moreover, in contrast, our study failed to find economic and statistical significance that OVX will impact the volatility of stock markets after considering the effect of VIX. In particular, based on both In-sample and Out-of-sample analysis, at least for daily, weekly, semimonthly and monthly periods, we may shed some doubts on that oil price uncertainty will influence the volatility of stock markets. Recently, from the perspectives of in-sample and out-of-sample investigations, [29] find an interesting result that oil market uncertainty fail to forecast the stock returns they selected, which seems similar to this study. There are two main differences between this study and theirs. Firstly, they focus on the impact of oil market uncertainty on stock returns while we concentrate that impact on volatility, that somehow makes the two studies complementary. Secondly, their conclusion is straightforward. Specifically, they just consider the connection between oil market uncertainty and stock returns, no any other factors are included while our models include another important factor (i.e., VIX) and our results imply that U.S. stock market factor is non-negligible. At last, both [28, 29] do not consider the effect of longer lagged external information while we find that can improve the volatility forecasting performance.

The rest of this study is organized as follow: section 2 outlines the data and methodology of In-sample and Out-of-sample. Section 3 is consisted of empirical analysis and robust check. Section 4 concludes.

## 2. Methodology and data

### 2.1. Methodology

To measure the volatility, we employ 5-minutes intraday data to obtain daily realized volatility (RV) of stock markets as follow ([30] points out that little evidence show that 5-minutes RV can be outperformed by other (even more sophisticated) models in terms of volatility measurement):

$${RV}_{t}=\sqrt{\sum_{i=1}^{m}{r_{t,i}}^{2}}$$ (1)

Where *r*_*t*,*i*_ denotes the log return during the period *i* in day *t*, *m* denotes frequency.

#### 2.1.1. In-sample

In this study, we choose Heterogeneous Autoregressive model (HAR) proposed by [31] to forecast the volatility of stock market as HAR is parsimonious and effective. The volatility forecasting models consisted of Generalized Autoregressive conditional heteroskedasticity model (GARCH) and Stochastic volatility model (SV) are prevalent but existing literatures show HAR-type is the better model for RV forecasting (see [32], for instance). The (benchmark) HAR model assumes the future volatility is driven by daily, weekly and monthly components which respectively represent the effects of Short-term, Middle-term and Long-term traders. To examine the impact of OVX and VIX on the volatility of stock markets, our empirical models are formalized as

$$\mathrm{HAR}:\;\log{RV}_{t+1}=c_{0}+\beta_{d}\log{RV}_{t}^{d}+\beta_{w}\log{RV}_{t}^{w}+\beta_{m}\log{RV}_{t}^{m}+\varepsilon_{t,h}$$ (2)

$$\mathrm{HARO}:\;\log{RV}_{t+1}=\beta^{o}X_{t}+\beta_{d,o}^{o}\log{OVX}_{t}^{d}+\beta_{w,o}^{o}\log{OVX}_{t}^{w}+\beta_{m,o}^{o}\log{OVX}_{t}^{m}+\varepsilon_{t,h}^{o}$$ (3)

$$\mathrm{HARV}:\;\log{RV}_{t+1}=\beta^{v}X_{t}+\beta_{d,v}^{v}\log{VIX}_{t}^{d}+\beta_{w,v}^{v}\log{VIX}_{t}^{w}+\beta_{m,v}^{v}\log{VIX}_{t}^{m}+\varepsilon_{t,h}^{v}$$ (4)

$$HAROV:\;log{RV}_{t+1}=\beta^{ov}X_{t}+\beta_{d,o}^{ov}log{OVX}_{t}^{d}+\beta_{w,o}^{ov}log{OVX}_{t}^{w}+\beta_{m,o}^{ov}log{OVX}_{t}^{m}$$

$$+\beta_{d,v}^{ov}\log{VIX}_{t}^{d}+\beta_{w,v}^{ov}\log{VIX}_{t}^{w}+\beta_{m,v}^{ov}\log{VIX}_{t}^{m}+\varepsilon_{t,h}^{ov}$$ (5)

Where $X_{t}={\left(1,log{RV}_{t}^{d},log{RV}_{t}^{w},log{RV}_{t}^{m}\right)}^{\prime },{RV}_{t}^{w}=\sqrt{\frac{1}{5}\sum_{i=0}^{4}{RV}_{t-i}^{2}},\beta^{*}=(c_{0,*}^{*},\beta_{d,*}^{*},\beta_{w,*}^{*},\beta_{m,*}^{*}),where*=(o,v,ov)$; $log\sigma_{t}^{d}=log\sigma_{t},log\sigma_{t}^{w}=log(\frac{1}{5}\sum_{i=0}^{4}\sigma_{t-i})$, $log\sigma_{t}^{m}=log(\frac{1}{22}\sum_{i=0}^{21}\sigma_{t-i})$, *σ*_*t*_∈(*OVX*_*t*_, *VIX*_*t*_). Then the magnitude and statistical significance of β* can be used to assess the impact of oil price uncertainty and U.S. stock uncertainty on the international stock market volatilities. The log transformation eliminates signs restriction of coefficients and that also will make the series more closed to normal distribution [33].

It worth noting that we use OVX and/or VIX of previous day, week and month (i.e. $log{OVX}_{t}^{d},log{OVX}_{t}^{w},log{OVX}_{t}^{m};log{VIX}_{t}^{d},log{VIX}_{t}^{w},log{VIX}_{t}^{m}$) rather than of only the previous day (i.e. commonly used $log{OVX}_{t}^{d},log{VIX}_{t}^{d}$). That because the information transmission between different markets which further induce volatility spillover takes time and maybe more than one day [34, 35]. In the section of empirical analysis, we will provide evidence that this model specification outperforms the commonly used one.

#### 2.1.2. Out-of-sample

For the out-of-sample process, we employ expanding window method to obtain the volatility forecasts values. Let $\hat{log{RV}_{t,k}}$ denotes the predicted value of log*RV*_*t*_ based on model *k*, *k* = (HAR, HARO, HARV, HAROV). The forecast error of model *k* at time *t* is *e*_*t*,*k*_, then $e_{t,k}=log{RV}_{t}-\hat{log{RV}_{t,k}}$. We use the well-known mean square forecast errors (MSFE) to evaluate the forecasting performance of models and $MSFE(k)=\frac{1}{T-T_{0}}\sum_{t=T_{0}+1}^{T}{e_{t,k}}^{2}$, where *T*_0_ denotes the number of data that used to obtain the first $\hat{log{RV}_{t,k}}$. The smaller MSFE implies the better model. Following previous researches [36–39], we test whether both OVX and VIX can (simultaneously) significantly improve the forecasting performance of log*RV*_*t*_ through [40] test, which is designed for the comparation of forecast performance of nested models. The Null hypothesis is that MSFE(*k*_1_)≤MSFE(*k*_2_), the alternative hypothesis is MSFE(*k*_1_)>MSFE(*k*_2_), (*k*_1_, *k*_2_)∈{(HAR, HARO), (HAR, HARV), (HARO, HAROV), (HARV, HAROV)}. The way to compute the MSFE-adjusted t-statistic is based on

$$f_{t}={\left(log{RV}_{t}-\hat{log{RV}_{t,bench}}\right)}^{2}-{\left(log{RV}_{t}-\hat{log{RV}_{t,model}}\right)}^{2}+{\left(\hat{log{RV}_{t,bench}}-\hat{log{RV}_{t,model}}\right)}^{2}$$ (6)

Then the MSFE-adjusted t-statistic is the t-statistic from regression of *f*_*t*_ on a constant.

### 2.2. Data

From Oxford-Man Realized Library [41], We collected the daily realized volatility data (*RV*_*t*_) of six major stock indexes due to their importance in global markets. These are S&P500 for USA, Nikkei225 for Japan, EUR STOXX50 for European region, FTSE100 for UK, CAC40 for France, DAX for Germany. Most researches focus on U.S. stock market while the relationship between different markets depend on market size, institutional development and other country-specific characteristics [42], using international data will increase the robust power of our findings and then yield more relative reliable results. The daily data of OVX and VIX are downloaded from CBOE website. The sample period ranges from May 10, 2007 to September 30, 2019 as the first data of OVX was published on May 10, 2007. The length of whole sample is about 3000 for each index. The Oxford-Man Realized Library has already calculated the realized variance via 5-minutes high frequency data so we directly extract them (call them, for example, “*rv*”) and address “*rv*” via *RV* = (100^2^*252*rv)^1/2^ to annualize them in percentage terms, which makes *RV* have the same units with OVX and VIX. More specifically, for example, $log{RV}_{t}^{w}=log{\left(100^{2}*252*(\frac{1}{5}\sum_{i=1}^{5}{rv}_{t+i})\right)}^{1/2}$, [2] and [43] address RV in similar way.

## 3. Empirical results

### 3.1. In-sample results

The full sample estimation results of HARO, HARV and HAROV presented in Table 1 are based on Eq (3), (4) and (5), respectively. For the regression results of HARO (second to fourth column), we can find all $\beta_{d,o}^{o}$ and $\beta_{w,o}^{o}$ are highly statistically significant. That means the OVX of previous day and week can significantly impact the current stock volatility of all indexes. This is not novel and is consistent with previous literature which provide evidence that oil market shows significant volatility spillover to stock markets. We then turn to the results of VIX (fifth to seventh column), almost all $\beta_{d,v}^{v},\beta_{w,v}^{v}$ and $\beta_{m,v}^{v}$ are highly statistically significant. That means the VIX of previous day, week, month can significantly impact the current stock volatility of all indexes. It is also consistent with the well-known conclusion that U.S. stock market would show volatility spillover to other country stock markets. Moreover, we can find the results of HARV are similar to that of HARO and the magnitude of the coefficients of OVX and VIX are comparable although the latter are larger. Unsurprisingly, the results of HARO and HARV imply that both OVX and VIX can significantly impact the realized volatility of all stock indexes if do not control the effects of each other. In other words, they can separately exert their influence on stock market volatility. This raises one of the main goals of this study: Investigating whether both of them can simultaneously work for volatility forecasting. We construct HAROV to this end and the results imply interesting conclusions. We can find $\beta_{d,v}^{ov}$ are still highly significant in all cases while all coefficients of OVX are insignificant except $\beta_{d,o}^{ov}$ of FTSE100 (at 10% significance level). Moreover, the magnitude of coefficients of VIX are much larger than that of OVX in all cases. That means OVX cannot offer additional significant information for volatility forecasting when the models have incorporated VIX while VIX effects are still considerable. Base on this, we speculate that the impact of OVX is negligible after controlling the effects of VIX.

**Table 1:** Full sample estimations of HARO, HARV and HAROV.

Indexes	HARO(OVX)			HARV(VIX)			HAROV (OVX and VIX)					
	daily	weekly	monthly	daily	weekly	monthly	daily	weekly	monthly	daily	weekly	monthly
	$\beta_{d,o}^{o}$	$\beta_{w,o}^{o}$	$\beta_{m,o}^{o}$	$\beta_{d,v}^{v}$	$\beta_{w,v}^{v}$	$\beta_{m,v}^{v}$	$\beta_{d,o}^{ov}$	$\beta_{w,o}^{ov}$	$\beta_{m,o}^{ov}$	$\beta_{d,v}^{ov}$	$\beta_{w,v}^{ov}$	$\beta_{m,v}^{ov}$
SP500	1.0451	-1.0294	0.0394	1.6764	-0.8773	-0.3139	0.1093	-0.0971	-0.0181	1.6466	-0.8587	-0.2962
	(0.000)	(0.000)	(0.661)	(0.000)	(0.000)	(0.000)	(0.433)	(0.596)	(0.846)	(0.000)	(0.000)	(0.002)
Nikkei225	0.93	-0.8174	-0.0676	1.1504	-0.7926	-0.2363	0.0875	-0.0908	-0.0215	1.1253	-0.7726	-0.2156
	(0.000)	(0.000)	(0.394)	(0.000)	(0.000)	(0.000)	(0.432)	(0.540)	(0.784)	(0.000)	(0.000)	(0.002)
EUR STOXX50	1.0368	-1.0415	0.0779	1.3121	-0.9725	-0.1889	0.1543	-0.2046	0.0907	1.2625	-0.9029	-0.2257
	(0.000)	(0.000)	(0.375)	(0.000)	(0.000)	(0.008)	(0.238)	(0.245)	(0.335)	(0.000)	(0.000)	(0.007)
FTSE100	1.0867	-0.9173	-0.0645	1.3422	-0.7184	-0.3089	0.2064	-0.0968	-0.0207	1.2844	-0.6679	-0.3163
	(0.000)	(0.000)	(0.414)	(0.000)	(0.000)	(0.000)	(0.087)	(0.537)	(0.806)	(0.000)	(0.000)	(0.000)
CAC40	0.9031	-0.8886	0.0355	1.1998	-0.9463	-0.1155	0.1478	-0.1301	0.0077	1.1568	-0.9095	-0.1187
	(0.000)	(0.000)	(0.624)	(0.000)	(0.000)	(0.048)	(0.177)	(0.340)	(0.919)	(0.000)	(0.000)	(0.071)
DAX	0.7492	-0.6978	0.0098	1.1115	-0.8756	-0.1175	0.0358	0.0056	1.00E-04	1.1025	-0.8704	-0.1289
	(0.000)	(0.000)	(0.889)	(0.000)	(0.000)	(0.043)	(0.763)	(0.969)	(0.999)	(0.000)	(0.000)	(0.054)

Notes: The results are based on the following models: HARO: $log{RV}_{t+1}=\beta^{o}X_{t}+\beta_{d,o}^{o}log{OVX}_{t}^{d}+\beta_{w,o}^{o}log{OVX}_{t}^{w}+\beta_{m,o}^{o}log{OVX}_{t}^{m}+\varepsilon_{t,h}^{o}$, HARV: $log{RV}_{t+1}=\beta^{v}X_{t}+\beta_{d,v}^{v}log{VIX}_{t}^{d}+\beta_{w,v}^{v}log{VIX}_{t}^{w}+\beta_{m,v}^{v}log{VIX}_{t}^{m}+\varepsilon_{t,h}^{v}$ and HAROV: $log{RV}_{t+1}=\beta^{ov}X_{t}+\beta_{d,o}^{ov}log{OVX}_{t}^{d}+\beta_{w,o}^{ov}log{OVX}_{t}^{w}+\beta_{m,o}^{ov}log{OVX}_{t}^{m}+\beta_{d,v}^{ov}log{VIX}_{t}^{d}+\beta_{w,v}^{ov}log{VIX}_{t}^{w}+\beta_{m,v}^{ov}log{VIX}_{t}^{m}+\varepsilon_{t,h}^{ov}$. The values in parenthesis denote the p-values of coefficients based on Newey-West adjustment. Columns two to four, five to seven and eight to thirteen denote the coefficients estimation of HARO, HARV and HAROV, respectively.

It is an interesting finding as many researches document the significant volatility spillover from oil market to stock markets, while Table 1 seems imply the “opposite” conclusion. It may imply that the information of OVX is included in VIX in terms of volatility forecasting of stock indexes. A plausible reason is the information of oil market is partly overlapped with that of U.S. stock market. As we know, the U.S. stock market, a global benchmark stock market, is consisted of several sectors such as oil-related sector, financials sector, consumer service sector, health care sector, among others. The VIX, an integrated volatility index used to represent U.S. stock market volatility, has incorporated the information of all sectors, including that of oil naturally. That causes VIX incorporates information of oil market in part. Consequently, the predictive power of OVX is weakened by VIX. On the other hand, the predictive power of VIX is weaken by OVX either (for example, the $\beta_{d,v}^{v}$ of SP500 decreases from 1.6764 in HARV to 1.6466 in HAROV), but the extent seems negligible and do not change the significance of the coefficients of VIX. That seems imply most information of VIX is not overlapped with OVX and a plausible explanation is VIX contains not only the information of oil market but also the volatility information of other sectors (such as financials; consumer service; health care; among others) which is not contained by OVX. Therefore, if the economic system of the country not relatively heavily depends on oil, OVX seems would not provide additional (significant) information beyond VIX with respect to volatility spillover. We must point out that we do not choose typical oil-exporting countries such as Middle east and African countries due to the high-frequency data availability limitation. In those countries, we may expect OVX will provide additional significant information beyond VIX for volatility spillover. That because oil exports account for a large fraction of trade in the oil-exporting countries, which results the stock markets of these countries are heavily connected with oil market [44]. In this sense, OVX seems more capable for stock volatility forecasting of these countries. Moreover, based on daily data and adjusted GARCH models, [28] has already provided the In-sample evidence that the impacts of OVX on the stock volatility of most Middle east and African countries are significant, even after controlling the effects of VIX. Due to the facts that out-of-sample evidence maybe more important [45] and In-sample evidence do not ensure Out-of-sample predictivity [46–48], the Out-of-sample evidence of [28] deserve further attention in the future.

It should be noted that the volatility of stock markets is not only influenced by implied volatility indexes (i.e., OVX and VIX) of previous day but also influenced by implied volatility indexes of previous week and/or month. We can observe daily and weekly coefficients of OVX or VIX are highly significant in all cases. Many researches of volatility forecasting [6, 7, 28] only focus on the information of previous day and our results imply the information of previous week and/or month should be taken into account for volatility forecasting. We confirm this further in the Out-of-sample section. In fact, the investors may react to the information diffusion at different times, which may result in the impact of new information on stock markets lasts a relative long time [34, 35].

Table 2 presents the adjusted R-square of the four models we selected. Both adjusted R-square of HARO and HARV is larger than the benchmark HAR model but the differences between R-square of HARV and HAROV are really insignificant which implies the impact of OVX is unconsidered after controlling VIX. On the other hand, the differences between R-square of HARO and HAROV are considerable which implies the impact of VIX is nonnegligible even after controlling the effects of OVX. All in all, the In-sample evidence suggest, although the impacts of OVX on RV of stock indexes seem economically and statistically significant, the effects vanish when controlling the effects of VIX while the information of VIX is effective and non-negligible.

**Table 2:** Adjusted R-square of HAR, HARO, HARV and HAROV (in percentage terms).

Indexes	HAR	HARO	HARV	HAROV
SP500	73.28	73.93	77.31	77.3
Nikkei225	64.77	65.81	68.99	68.98
EUR STOXX50	62.31	63.34	66.43	66.45
FTSE100	65.42	66.73	70.78	70.95
CAC40	71.1	71.97	74.7	74.7
DAX	71.37	71.98	74.31	74.33

### 3.2. Out-of-sample analysis

#### 3.2.1. Out-of-sample evidence of the impact of OVX and VIX on stock volatility

We derive 2000 out-of-sample forecasts of $\hat{log{RV}_{t+1,k}}$ of each index and the results are presented in Table 3. MSFE(HAR), MSFE(HARO), MSFE(HARV), MSFE(HAROV) are mean square forecast errors derived from Eq (2), (3), (4), (5), respectively. Both In-sample period and Out-of-sample period are long enough to derive a reliable and meaningful statistic inference [2]. At first glance, again, OVX can separately significantly improve the volatility forecasting of all indexes as P1 reject the null hypothesis that MSFE(HARO)≥MSFE(HAR) in all cases. The effects of OVX are weakened to disappear when controlling the effects of VIX as P4 fail to reject the null hypothesis that MSFE(HAROV)≥MSFE(HARV) in almost all cases. The only exception is the case of FTSE100, which is consistent with the result of In-sample analysis (in Table 1, $\beta_{d,o}^{ov}$ of FTSE100 in HAROV is still significant at 10% significance level), although the improvement derived from OVX is relatively small (from 0.792 to 0.789). Maybe a plausible explanation is the Brent oil futures traded in London makes the UK stock market more closely linked to oil market than other countries. We can observe that the difference between MSFE(HAROV) and MSFE(HARV) is really insignificant. Moreover, MSFE(HARV) are (a bit) smaller than MSFE(HAROV) in 5 out of 6 cases. This indicates that OVX provide negative information for volatility forecasting when the effects of VIX are already considered. In fact, if OVX do not offer additional information, incorporate OVX into the model may bring the noise to model which furtherly be harmful for model estimation and subsequent forecasts. On the other hand, P2 shows the rejections of null hypothesis of MSFE(HARV)≥MSFE(HAR) imply VIX can separately significantly improve the forecasts of volatility and the improvements derived from separate VIX are more marked than that derived from separate OVX. The results of P3 imply the improvements of forecast derived from the information of VIX are stable even after controlling the effects of OVX. In a summary, the results of Table 3 are consistent with that of In-sample evidences.

**Table 3:** Out-of-sample forecasts performance of HAR, HARO, HARV and HAROV.

Indexes	MSFE(HAR)	MSFE(HARO)	MSFE(HARV)	MSFE(HAROV)	P1	P2	P3	P4
SP500	0.1076	0.1053	0.0904	0.0909	0.0000	0.0000	0.0000	0.5584
Nikkei225	0.0871	0.0853	0.0773	0.0775	0.0000	0.0000	0.0000	0.9106
EUR STOXX50	0.1126	0.1098	0.1013	0.1016	0.0000	0.0000	0.0000	0.2989
FTSE100	0.0937	0.0907	0.0792	0.0789	0.0000	0.0000	0.0000	0.0010
CAC40	0.0663	0.0647	0.0591	0.0592	0.0000	0.0000	0.0000	0.2602
DAX	0.0688	0.0679	0.0627	0.0631	0.0000	0.0000	0.0000	0.4760

Notes: MSFE(HAR), MSFE(HARO), MSFE(HARV), MSFE(HAROV) are derived from Eq (2), (3), (4), (5), respectively. Conditional on [40], P1, P2, P3, P4 denote the p-values of hypothesis tests with null hypothesis of MSFE(HARO)≥MSFE(HAR), MSFE(HARV)≥MSFE(HAR), MSFE(HAROV)≥MSFE(HARO), MSFE(HAROV)≥MSFE(HARV), respectively.

The evidences mentioned above focus on the average of forecasts performance which only represent the results of the whole Out-of-sample period. We compute “Cumulate square forecast errors” (cumSFE) proposed by Rapach et al., 2013 [1], which aims to have a deep insight to the evolution of forecasting performance of competing model relative to benchmark model. Formally, in our framework,

$$cumSFE(j,N)=\sum_{t=T_{0}+1}^{N}\left({e_{t,HAR}}^{2}-{e_{t,j}}^{2}\right),\;j=(HARO,HARV,HAROV);$$ (7)

$$\forall N=T_{0}+1,T_{0}+2,\ldots ,T.$$

**Fig 1:**
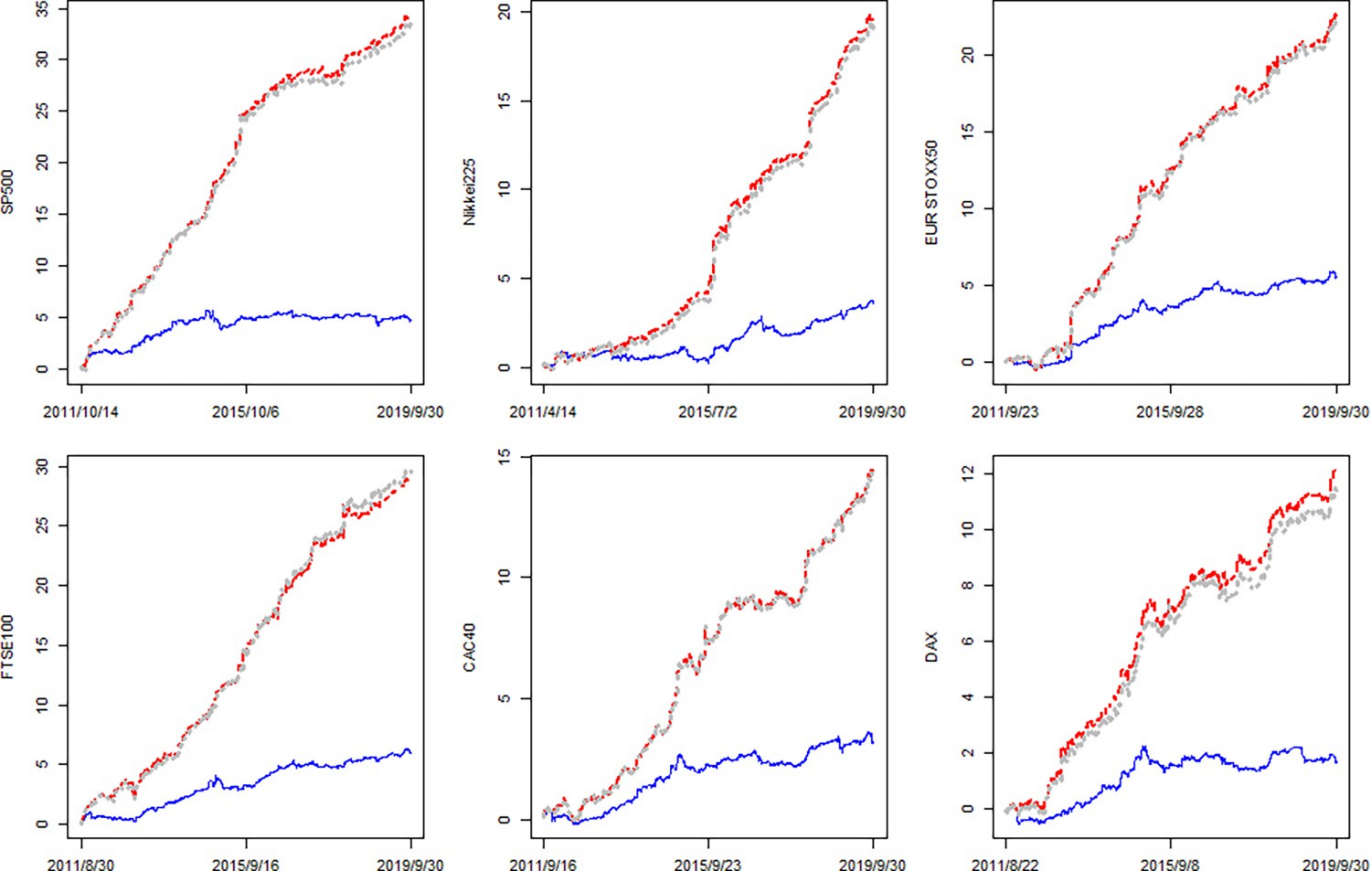
The cumSFE of HARO, HARV and HAROV relative to HAR. Notes: The blue line, red line and gray line denotes the cumSFE(*HARO*, *N*), cumSFE(*HARV*, *N*) and cumSFE(*HAROV*, *N*), respectively.

The cumSFE(*j*, *N*) can depict the dynamics of forecasting performance of competing model relative to benchmark model HAR and the (consistently) larger cumSFE imply the (consistently) better model. That further imply that whether our findings are robust over time. Base on Eq (7), results are provided in Fig 1. The blue lines, red lines and gray lines denotes the cumSFE(*HARO*, *N*), cumSFE(*HARV*, *N*) and cumSFE(*HAROV*, *N*), respectively. We can observe some evidences about the model forecasting performances. Firstly, all blue and red lines generally lay above the zero line and are upward despite some small fluctuations. That imply OVX and VIX can separately provide additional predictive power for international stock markets volatility forecasting. Secondly, the gray lines always lay very closed to (even lower than) red line over time in all cases. That means HAROV consistently performs very closed to (even worse than) HARV, which imply OVX fails to provide positive additional information when VIX has been incorporated into the model. Thirdly, the gray line consistently lay above the blue line and the differences between them are more marked over time in all cases. That means HAROV consistently outperform HARO, which imply VIX can provide consistently effective information even after controlling OVX. Last but not least, as time changes, the fluctuations of gray line (cumSFE(HAROV)) is highly consistent with that of Red line (cumSFE(HARV)), which also indicate VIX dominates OVX in HAROV and the role of OVX seems negligible. All in all, Fig 1 confirm our conclusions from the perspective of dynamics.

In summary, Out-of-sample estimations provide evidence that OVX and VIX seems will not simultaneously work in volatility forecasting. Specifically, separately utilizing OVX can significantly improve the RV forecasting for all indexes we study but the improvements vanish after controlling the effects of VIX while VIX provide consistently effective information even after controlling OVX. This conclusion of Out-of-sample is consistent with that of In-sample.

#### 3.2.2. Out-of-sample evidences of the longer lagged effects

As mentioned in section 3.1, many researches of volatility forecasting only focus on the information of previous day and their frameworks are similar to

$$\mathrm{HAROd}:\;\log{RV}_{t+1}=\beta X_{t}+\beta_{d,o}^{o}\log{OVX}_{t}^{d}+\varepsilon_{t,h}$$ (8)

$$\mathrm{HARVd}:\;\log{RV}_{t+1}=\beta X_{t}+\beta_{d,v}^{v}\log{VIX}_{t}^{d}+\varepsilon_{t,h}$$ (9)

$$\mathrm{HAROVd}:\;\log{RV}_{t+1}=\beta X_{t}+\beta_{d,o}^{ov}\log{OVX}_{t}^{d}+\beta_{d,v}^{ov}\log{VIX}_{t}^{d}+\varepsilon_{t,h}$$ (10)

Where HAROd denotes only previous day of OVX is considered. The In-sample results (Table 1) documents that the coefficients of previous week (even previous month) of OVX or VIX are significant. Accordingly, we would expect longer lagged effects will improve the Out-of-sample forecasting. In this section, we will test the hypothesis that HAROd, HARVd, HAROVd (Eq (8), (9), (10)) cannot be outperformed by HARO, HARV, HAROV (Eq (3), (4), (5)), respectively. Note that HAROd is nested in HARO, so the test of [40] is suitable here either. Table 4 presents MSFE(HAROd), MSFE(HARVd), MSFE(HAROVd) and MSFE(HARO), MSFE(HARV), MSFE(HAROV) along with the p-values of corresponding hypothesis tests.

**Table 4:** MSFE(HAROd), MSFE(HARVd), MSFE(HAROVd) and MSFE(HARO), MSFE(HARV), MSFE(HAROV) along with the p-values of corresponding hypothesis tests.

Indexes	HAROd	HARVd	HAROVd	HARO	HARV	HAROV
SP500	0.1077	0.0949	0.0949	0.1053	0.0904	0.0909
	(0.0000)	(0.0000)	(0.0000)			
Nikkei225	0.0873	0.0839	0.0837	0.0853	0.0773	0.0775
	(0.0000)	(0.0000)	(0.0000)			
EUR STOXX50	0.1122	0.1087	0.1088	0.1098	0.1013	0.1016
	(0.0000)	(0.0000)	(0.0000)			
FTSE100	0.0928	0.0838	0.0833	0.0907	0.0792	0.0789
	(0.0000)	(0.0000)	(0.0000)			
CAC40	0.0663	0.0643	0.0644	0.0647	0.0591	0.0592
	(0.0000)	(0.0000)	(0.0000)			
DAX	0.0688	0.0671	0.0671	0.0679	0.0627	0.0631
	(0.0000)	(0.0000)	(0.0000)			

Notes: Conditional on [40], values in parathesis denote the p-values of hypothesis tests with null hypothesis of HAROd, HARVd, HAROVd cannot be outperformed by HARO, HARV, HAROV, respectively.

We can observe that no matter for HAROd, HARVd or HAROVd, the hypotheses of that they cannot be outperformed by HARO, HARV, HAROV are rejected in all cases. That imply the volatility forecasting performance of models which only consider daily effect can be improved by incorporating longer lagged length. A plausible explanation is the investors may react to the information diffusion at different times, which may result in the impact of new information on stock markets lasts a relative long time [34, 35]. This section validates our model settings from Out-of-sample perspective.

### 3.3. Volatility forecasting for long horizons

This section extends the forecasting horizon from one day to long horizon and the forecasting models are given by:

$$\mathrm{LHAR}:\;\log{RV}_{h|t}=c_{0}+\beta_{d}\log{RV}_{t}^{d}+\beta_{w}\log{RV}_{t}^{w}+\beta_{m}\log{RV}_{t}^{m}+\varepsilon_{t,h}$$ (11)

$$\mathrm{LHARO}:\;\log{RV}_{h|t}=\beta^{o}X_{t}+\beta_{d,o}^{o}\log{OVX}_{t}^{d}+\beta_{w,o}^{o}\log{OVX}_{t}^{w}+\beta_{m,o}^{o}\log{OVX}_{t}^{m}+\varepsilon_{t,h}^{o}$$ (12)

$$\mathrm{LHARV}:\;\log{RV}_{h|t}=\beta^{v}X_{t}+\beta_{d,v}^{v}\log{VIX}_{t}^{d}+\beta_{w,v}^{v}\log{VIX}_{t}^{w}+\beta_{m,v}^{v}\log{VIX}_{t}^{m}+\varepsilon_{t,h}^{v}$$ (13)

$$LHAROV:\;log{RV}_{h|t}=\beta^{ov}X_{t}+\beta_{d,o}^{ov}log{OVX}_{t}^{d}+\beta_{w,o}^{ov}log{OVX}_{t}^{w}+\beta_{m,o}^{ov}log{OVX}_{t}^{m}$$

$$+\beta_{d,v}^{ov}\log{VIX}_{t}^{d}+\beta_{w,v}^{ov}\log{VIX}_{t}^{w}+\beta_{m,v}^{ov}\log{VIX}_{t}^{m}+\varepsilon_{t,h}^{ov}$$ (14)

Where $log{RV}_{h|t}=log{\left(\frac{1}{h}\sum_{i=1}^{h}{RV}_{t+i}^{2}\right)}^{1/2}$. The only differences between Eq (3–6) and Eqs (11–14) are the dependent variables (i.e. log*RV*_*t*+1_ vs. log*RV*_*h*|*t*_). In this study, h = 5, 10, 22 which means the weekly, biweekly and monthly volatility forecasting. It is obvious log*RV*_*t*+1_ is a special case of log*RV*_*h*|*t*_ (i.e. h = 1).

We first conduct the In-sample analysis and results are shown in Table 5. We can find the impacts of OVX and VIX on stock volatility changes over horizons. Generally, the magnitude and significance of the coefficients decrease with the increase of horizon. That may because the response of stock market activities to other markets shocks will complete in a certain time period [49]. However, our main conclusions still hold true. We can find $\beta_{d,o}^{o}$ in LHARO (second column) and $\beta_{d,v}^{v}$ in LHARV (fifth column) are significant in all cases, even for the horizon of 22 days. That imply OVX and VIX can separately impact the future stock volatilities. What count is the $\beta_{d,o}^{ov}$ in LHAROV are insignificant while $\beta_{d,v}^{ov}$ in LHAROV are still significant in all cases. That imply OVX is unable to provide additional information for stock volatility forecasting when the model has incorporated VIX, while the impacts of VIX are considerable even after controlling OVX effects.

**Table 5:** Full sample estimations of LHARO, LHARV and LHAROV (long forecasting horizons).

Indexes	LHARO			LHARV			LHAROV					
	daily	weekly	monthly	daily	weekly	monthly	daily	weekly	monthly	daily	weekly	monthly
	$\beta_{d,o}^{o}$	$\beta_{w,o}^{o}$	$\beta_{m,o}^{o}$	$\beta_{d,v}^{v}$	$\beta_{w,v}^{v}$	$\beta_{m,v}^{v}$	$\beta_{d,o}^{ov}$	$\beta_{w,o}^{ov}$	$\beta_{m,o}^{ov}$	$\beta_{d,v}^{ov}$	$\beta_{w,v}^{ov}$	$\beta_{m,v}^{ov}$
	*h* = 5											
SP500	0.8194	-0.8591	0.1072	1.3347	-0.5888	-0.2031	0.0721	-0.1193	0.0444	1.3116	-0.5541	-0.2142
	(0.000)	(0.000)	(0.476)	(0.000)	(0.000)	(0.120)	(0.605)	(0.556)	(0.752)	(0.000)	(0.002)	(0.161)
Nikkei225	0.7597	-0.6611	-0.0375	0.9606	-0.633	-0.1751	0.0398	-0.0619	-0.0042	0.949	-0.6175	-0.162
	(0.000)	(0.001)	(0.784)	(0.000)	(0.000)	(0.062)	(0.770)	(0.779)	(0.976)	(0.000)	(0.000)	(0.136)
EUR STOXX50	0.8677	-0.9474	0.1683	1.0348	-0.7588	-0.1076	0.1818	-0.3078	0.1806	0.9726	-0.6478	-0.1803
	(0.000)	(0.000)	(0.267)	(0.000)	(0.000)	(0.262)	(0.153)	(0.149)	(0.293)	(0.000)	(0.000)	(0.135)
FTSE100	0.891	-0.8141	0.0415	1.0752	-0.5734	-0.1771	0.1946	-0.1592	0.065	1.0163	-0.4935	-0.2179
	(0.000)	(0.000)	(0.712)	(0.000)	(0.000)	(0.046)	(0.055)	(0.290)	(0.592)	(0.000)	(0.000)	(0.041)
CAC40	0.7183	-0.7595	0.1015	0.9101	-0.707	-0.053	0.1525	-0.1971	0.0798	0.8618	-0.642	-0.0844
	(0.000)	(0.000)	(0.429)	(0.000)	(0.000)	(0.541)	(0.152)	(0.231)	(0.569)	(0.000)	(0.000)	(0.403)
DAX	0.5653	-0.5185	0.0308	0.7778	-0.5552	-0.0897	0.0678	-0.0552	0.0446	0.7577	-0.5262	-0.1196
	(0.000)	(0.001)	(0.811)	(0.000)	(0.000)	(0.322)	(0.537)	(0.749)	(0.764)	(0.000)	(0.000)	(0.282)
	*h* = 10											
SP500	0.4776	-0.4733	0.0745	1.069	-0.3563	-0.1266	-0.1562	0.158	-0.0015	1.1132	-0.3917	-0.1366
	(0.001)	(0.106)	(0.744)	(0.000)	(0.098)	(0.564)	(0.320)	(0.630)	(0.995)	(0.000)	(0.107)	(0.587)
Nikkei225	0.5203	-0.383	-0.0662	0.7536	-0.4574	-0.132	-0.0829	0.1051	-0.0443	0.781	-0.4909	-0.1119
	(0.000)	(0.100)	(0.725)	(0.000)	(0.003)	(0.319)	(0.529)	(0.684)	(0.825)	(0.000)	(0.005)	(0.486)
EUR STOXX50	0.5804	-0.6212	0.1477	0.8168	-0.5261	-0.1034	-0.0027	-0.1017	0.1759	0.8085	-0.4613	-0.1909
	(0.000)	(0.007)	(0.493)	(0.000)	(0.001)	(0.534)	(0.984)	(0.706)	(0.471)	(0.000)	(0.016)	(0.340)
FTSE100	0.615	-0.5272	0.0459	0.861	-0.3673	-0.1534	0.0245	0.0099	0.0813	0.8502	-0.3218	-0.2119
	(0.000)	(0.004)	(0.779)	(0.000)	(0.033)	(0.349)	(0.837)	(0.961)	(0.607)	(0.000)	(0.085)	(0.236)
CAC40	0.4929	-0.4949	0.0752	0.7012	-0.4487	-0.0866	0.0273	-0.0651	0.0861	0.689	-0.4117	-0.1314
	(0.000)	(0.009)	(0.681)	(0.000)	(0.004)	(0.599)	(0.820)	(0.775)	(0.680)	(0.000)	(0.016)	(0.469)
DAX	0.4045	-0.2966	-0.014	0.597	-0.2986	-0.1501	-0.0046	0.0328	0.0458	0.5987	-0.288	-0.1894
	(0.001)	(0.124)	(0.938)	(0.000)	(0.049)	(0.334)	(0.969)	(0.891)	(0.833)	(0.000)	(0.097)	(0.289)
	*h* = 22											
SP500	0.5026	-0.3276	-0.0839	0.9236	-0.3283	-0.0716	-0.0331	0.2386	-0.1879	0.945	-0.418	-0.0079
	(0.000)	(0.355)	(0.785)	(0.000)	(0.221)	(0.773)	(0.828)	(0.512)	(0.544)	(0.000)	(0.166)	(0.978)
Nikkei225	0.5425	-0.1728	-0.2772	0.6844	-0.2623	-0.2878	-0.001	0.2327	-0.1929	0.6941	-0.3523	-0.2348
	(0.001)	(0.615)	(0.366)	(0.000)	(0.283)	(0.191)	(0.995)	(0.543)	(0.557)	(0.000)	(0.168)	(0.329)
EUR STOXX50	0.5901	-0.5263	0.0796	0.7183	-0.3237	-0.1926	0.0662	-0.1149	0.1592	0.6909	-0.2523	-0.282
	(0.000)	(0.049)	(0.757)	(0.000)	(0.107)	(0.357)	(0.586)	(0.684)	(0.564)	(0.000)	(0.255)	(0.256)
FTSE100	0.5811	-0.4687	0.0433	0.7865	-0.2562	-0.255	0.0212	-0.0099	0.136	0.7738	-0.1884	-0.3415
	(0.000)	(0.067)	(0.848)	(0.000)	(0.247)	(0.249)	(0.865)	(0.971)	(0.573)	(0.000)	(0.415)	(0.170)
CAC40	0.4941	-0.453	0.0594	0.6444	-0.3154	-0.1567	0.0541	-0.0892	0.1143	0.6232	-0.2629	-0.2202
	(0.000)	(0.060)	(0.800)	(0.000)	(0.089)	(0.437)	(0.643)	(0.732)	(0.642)	(0.000)	(0.168)	(0.295)
DAX	0.4352	-0.2831	-0.0412	0.5702	-0.1906	-0.2374	0.0343	-0.003	0.0653	0.5603	-0.165	-0.2884
	(0.000)	(0.207)	(0.850)	(0.000)	(0.275)	(0.279)	(0.772)	(0.990)	(0.778)	(0.000)	(0.363)	(0.204)

Notes: The results are based on the following models: LHARO: $log{RV}_{h|t}=\beta^{o}X_{t}+\beta_{d,o}^{o}log{OVX}_{t}^{d}+\beta_{w,o}^{o}log{OVX}_{t}^{w}+\beta_{m,o}^{o}log{OVX}_{t}^{m}+\varepsilon_{t,h}^{o}$, LHARV: $log{RV}_{h|t}=\beta^{v}X_{t}+\beta_{d,v}^{v}log{VIX}_{t}^{d}+\beta_{w,v}^{v}log{VIX}_{t}^{w}+\beta_{m,v}^{v}log{VIX}_{t}^{m}+\varepsilon_{t,h}^{v}$ and LHAROV: $log{RV}_{h|t}=\beta^{ov}X_{t}+\beta_{d,o}^{ov}log{OVX}_{t}^{d}+\beta_{w,o}^{ov}log{OVX}_{t}^{w}+\beta_{m,o}^{ov}log{OVX}_{t}^{m}+\beta_{d,v}^{ov}log{VIX}_{t}^{d}+\beta_{w,v}^{ov}log{VIX}_{t}^{w}+\beta_{m,v}^{ov}log{VIX}_{t}^{m}+\varepsilon_{t,h}^{ov}$. The values in parenthesis denote the p-values of coefficients based on Newey-West adjustment. Columns two to four, five to seven and eight to thirteen denote the coefficients estimation of LHARO, LHARV and LHAROV, respectively.

The adjusted R-square of LHAR, LHARO, LHARV and LHAROV are shown in Table 6. It is unsurprising that R-squares decrease with the increase of horizons and R-square of LHARO and LHARV are larger than that of LHAR. What count is the differences between R-square of LHARV and LHAROV are relatively insignificant while the differences between R-square of LHARO and LHAROV are considerable. The results of Table 6 are consistent with that of Table 5.

**Table 6:** Adjusted R-square of LHAR, LHARO, LHARV and LHAROV (in percentage terms).

Indexes	*h* = 5				*h* = 10				*h* = 22			
	LHAR	LHARO	LHARV	LHAROV	LHAR	LHARO	LHARV	LHAROV	LHAR	LHARO	LHARV	LHAROV
SP500	76.54	77.04	80.09	80.07	73.88	74.14	76.92	76.92	63.23	63.61	65.66	65.7
Nikkei225	69.29	70.27	73.57	73.57	67.57	68.27	71.06	71.05	49.75	51.11	53.04	53.19
EUR STOXX50	71.95	73.01	75.55	75.67	69.66	70.39	72.55	72.71	58.84	59.95	61.9	62.26
FTSE100	75.77	76.98	80.51	80.77	74.19	75.08	78.17	78.51	62.03	63.04	65.45	66.01
CAC40	76.92	77.63	79.59	79.63	74.13	74.58	76.18	76.24	63.92	64.55	66.07	66.25
DAX	77.42	77.94	79.4	79.47	75.1	75.57	76.78	76.92	65.84	66.5	67.74	67.99

Then we turn to the Out-of-sample analysis. Table 7 shows the results of hypothesis test of volatility forecasting performance comparations for long horizons. As we find in Table 5 (In-sample analysis), the predictive power of OVX and VIX tends to decrease with the increase of horizon as the p-values (P1 and P2) of hypothesis is larger in long horizon than that in short horizon. However, results of Table 7 do not contradict our conclusions. In most cases, values of P1 indicate the rejections of hypothesis that LHAR cannot be outperformed by LHARO while values of P4 indicate it’s unable to reject the hypothesis that LHARV cannot be outperformed by LHAROV. That imply OVX can separately significantly improve the stock volatility forecasting, but the improvements seem disappear after controlling the effects of VIX. On the other hand, the implication conditional on values of P2 and P3 is the effects of VIX are considerable, even after controlling OVX.

**Table 7:** Out-of-sample forecasts performance comparation of LHAR, LHARO, LHARV and LHAROV (long forecasting horizons).

Indexes	P1	P2	P3	P4
	*h* = 5			
SP500	0.0004	0.0000	0.0000	0.2564
Nikkei225	0.0011	0.0000	0.0000	0.8992
EUR STOXX50	0.0000	0.0000	0.0000	0.1307
FTSE100	0.0000	0.0000	0.0000	0.0241
CAC40	0.0000	0.0000	0.0000	0.1378
DAX	0.0012	0.0000	0.0000	0.3808
	*h* = 10			
SP500	0.0523	0.0000	0.0000	0.3022
Nikkei225	0.1214	0.0000	0.0000	0.7218
EUR STOXX50	0.0009	0.0000	0.0000	0.2471
FTSE100	0.0001	0.0000	0.0000	0.1321
CAC40	0.0086	0.0000	0.0000	0.2445
DAX	0.0198	0.0000	0.0000	0.3511
	*h* = 22			
SP500	0.0634	0.0000	0.0001	0.2888
Nikkei225	0.1141	0.0000	0.0002	0.5966
EUR STOXX50	0.0046	0.0000	0.0009	0.2545
FTSE100	0.0012	0.0000	0.0001	0.2016
CAC40	0.0334	0.0001	0.0068	0.2784
DAX	0.0685	0.0001	0.0090	0.3128

Notes: P1, P2, P3, P4 denote the p-values of hypothesis tests with null hypothesis of MSFE(LHARO)≥MSFE(LHAR), MSFE(LHARV)≥MSFE(LHAR), MSFE(LHAROV)≥MSFE(LHARO), MSFE(LHAROV)≥MSFE(LHARV), respectively.

**Fig 2:**
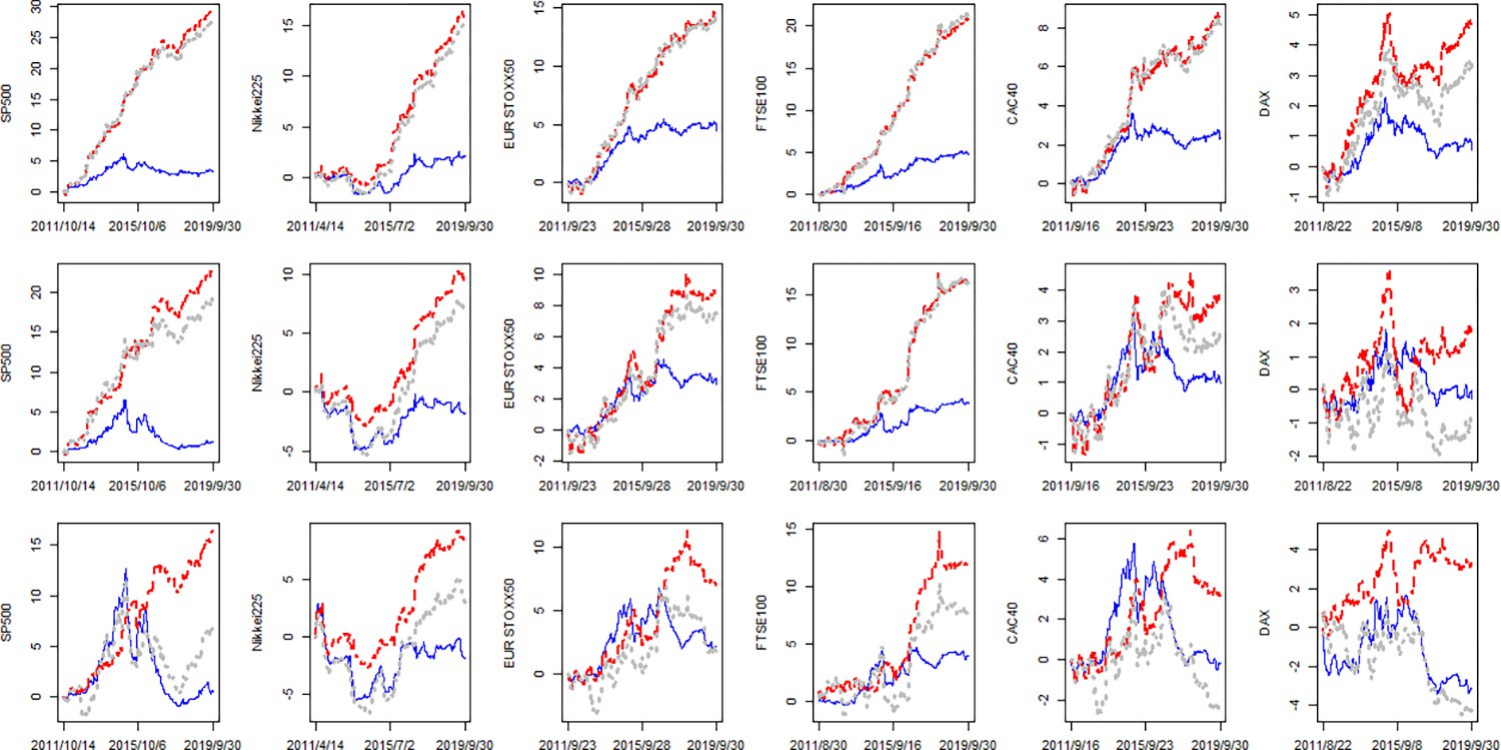
The cumSFE of LHARO, LHARV and LHAROV relative to LHAR. Notes: The first, second, third and row corresponding to *h* = 5,10,22, respectively. The blue line, red line and gray line denotes the cumSFE(*LHARO*, *N*), cumSFE(*LHARV*, *N*) and cumSFE(*LHAROV*, *N*), respectively.

Besides the average forecasting performance evaluation of long horizons, the dynamics of the volatility forecasting performance are presented in Fig 2. At first glance, the dynamics of long horizons are more fluctuated than that of daily forecasting and the fluctuations increase with the horizon. That imply long horizons volatility forecasting are more difficult. In some cases, we can find some models perform poorly as their corresponding lines lay below zero (especially for h = 22). However, Fig 2 do not oppose to our main conclusions either. In most cases, the blue lines and red lines lay above zero which indicate the separate predictive power of OVX and VIX. Moreover, the gray lines lay close to (even lower than) red lines but clearly above the blue lines, which again indicate the information of OVX is not positive for volatility forecasting after controlling the VIX while VIX effects are considerable even after controlling OVX. At last, the trajectories of gray lines are very similar to that of red lines show the dominated role of VIX in LHAROV while OVX seems redundant.

From the perspective of long horizons forecasting, we conduct the comparations of daily lagged models and long lagged models (all model settings are similar to that in section 3.2.2) to verify the superiority of the latter one. The results are presented in Table 8. The rejections of hypothesis in most cases imply long lagged effects should be considered.

**Table 8:** The tests of forecasting performance comparation between daily lagged and long lagged models (long horizons).

Indexes	*h* = 5			*h* = 10			*h* = 22		
	LHAROd	LHARVd	LHAROVd	LHAROd	LHARVd	LHAROVd	LHAROd	LHARVd	LHAROVd
SP500	0.000	0.000	0.000	0.011	0.031	0.076	0.123	0.491	0.612
Nikkei225	0.000	0.000	0.000	0.075	0.000	0.001	0.099	0.004	0.016
EUR STOXX50	0.000	0.000	0.000	0.002	0.000	0.001	0.013	0.019	0.097
FTSE100	0.000	0.000	0.000	0.002	0.013	0.018	0.008	0.075	0.127
CAC40	0.000	0.000	0.000	0.002	0.001	0.003	0.056	0.039	0.197
DAX	0.001	0.000	0.000	0.026	0.001	0.014	0.099	0.014	0.118

Notes: Values in parathesis denote the p-values of hypothesis tests with null hypothesis of LHAROd, LHARVd, LHAROVd cannot be outperformed by LHARO, LHARV, LHAROV, respectively.

Overall, the long forecasting horizons analysis do not overturn our model settings and findings.

The finds of this study can be good complementary addition to the existing literatures. On the one hand, many literatures have shown the significant volatility spillover from oil market to stock markets. For example, [4] shows oil volatility can be used as an effective factor to significantly improve the stock market volatility forecasting. By constructing oil market uncertainty through realized volatility of monthly oil prices, [5] provide similar results that oil market uncertainty also can provide predictive power for stock market volatility. Our study provides opposite conclusion that the effects of oil market uncertainty on stock markets volatility seems insignificant. The main reason might be ignorance of the volatility spillover effects from U.S stock market as our empirical evidences explicitly show the predictive power of oil market uncertainty vanish after controlling the volatility spillover from U.S stock market. Our study contributes the existing literatures by empirically show the insignificant impacts of oil market uncertainty on stock volatility forecasting and that will enrich the understanding of oil-stock nexus. On the other hand, [2] explicitly show that VIX can significantly improve the volatility forecasting performance of the international stock markets they selected. Recently, through decomposing the realized volatility into “good volatility” and “bad volatility”, [26] provide evidences that VIX will show significant predictive power for the “bad volatility” of Chinese stock market. Our study is like a further investigation. Part of our evidences support their findings that VIX can not be ignored with respect to stock volatility forecasting, what important is we furtherly explore the question that whether oil market uncertainty can be ignored. In our framework, one of the possible next steps of [26] could be the investigation of the predictive power of oil market uncertainty for Chinese stock market volatility.

### 3.4. Rolling window check

In this section, we check whether our conclusions are robust for rolling window method. [50, 51] shows different window sizes may result in different out-of-sample results and we choose different window sizes to this end. It should be noted that the window size selection is a trade-off between the initial in-sample length is long enough to precisely estimate the parameters and the Out-of-sample numbers should be desirable large for conducting meaningful forecasting evaluation. In this way, we set the out-of-sample lengths to 2000 and 1500, then the corresponding initial in-sample lengths are about 1000 and 1500. The initial In-sample lengths we selected are similar to that of [6]. The results of both window sizes are similar and for simplicity we only present the results of rolling window method with 2000 out-of-sample numbers here. Table 9 shows the p-values of hypotheses tests which are similar to Tables 3 and 7.

**Table 9:** Out-of-sample forecasts performance comparations of LHAR, LHARO, LHARV and LHAROV based on rolling window with 2000 out-of-sample numbers.

Indexes	P1	P2	P3	P4
		*h* = 1		
SP500	0.0000	0.0000	0.0000	0.5774
Nikkei225	0.0000	0.0000	0.0000	0.1176
EUR STOXX50	0.0000	0.0000	0.0000	0.3251
FTSE100	0.0000	0.0000	0.0000	0.0013
CAC40	0.0000	0.0000	0.0000	0.2962
DAX	0.0000	0.0000	0.0000	0.6926
		*h* = 5		
SP500	0.0012	0.0000	0.0000	0.2993
Nikkei225	0.0024	0.0000	0.0000	0.1609
EUR STOXX50	0.0000	0.0000	0.0000	0.0577
FTSE100	0.0000	0.0000	0.0000	0.0045
CAC40	0.0000	0.0000	0.0000	0.1125
DAX	0.0053	0.0000	0.0000	0.4995
		*h* = 10		
SP500	0.0667	0.0000	0.0000	0.3495
Nikkei225	0.0787	0.0000	0.0000	0.2182
EUR STOXX50	0.0015	0.0000	0.0000	0.1974
FTSE100	0.0000	0.0000	0.0000	0.0118
CAC40	0.0073	0.0000	0.0001	0.2628
DAX	0.0251	0.0000	0.0016	0.4472
		*h* = 22		
SP500	0.0460	0.0000	0.0102	0.3268
Nikkei225	0.0650	0.0022	0.0022	0.1538
EUR STOXX50	0.0080	0.0001	0.0514	0.3016
FTSE100	0.0002	0.0004	0.0113	0.0357
CAC40	0.0151	0.0002	0.1143	0.3277
DAX	0.0547	0.0009	0.0722	0.4227

Notes: Conditional on [40], P1, P2, P3, P4 denote the p-values of hypothesis tests with null hypothesis of MSFE(HARO)≥MSFE(HAR), MSFE(HARV)≥MSFE(HAR), MSFE(HAROV)≥MSFE(HARO), MSFE(HAROV)≥MSFE(HARV), respectively.

In Table 9, rejections of P1 (P2) imply that OVX (VIX) can separately provide additional information to the volatility forecasting when they are solely incorporated into the model. Failures of rejection of P4 imply OVX cannot improve the forecasts performance when VIX has been considered. On the contrary, rejections of P4 imply VIX can persistently facilitate volatility forecasting even after controlling the effects of OVX. Although there is a little difference between Table 9 and Table 3 along with 7, main differences are Table 9 reject FTSE100 for *h* = 10, 22 while Table 7 fail to reject that, the conclusions of Table 3 along with 7 is still supported by Table 9.

**Fig 3:**
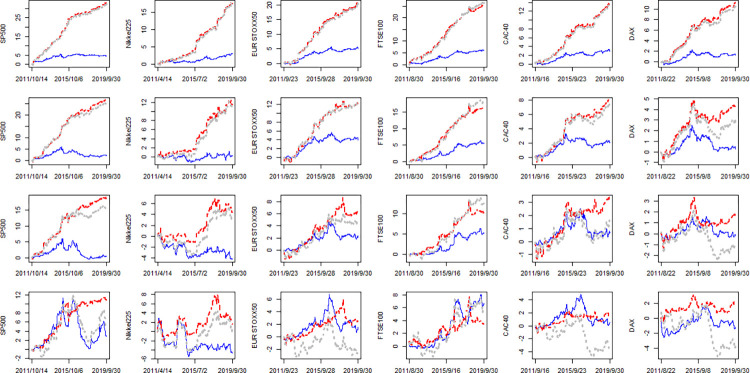
The cumSFE of LHARO, LHARV and LHAROV relative to LHAR (rolling window). Notes:The first, second, third and fourth row corresponding to *h* = 1,5,10,22, respectively. The blue line, red line and gray line denotes the cumSFE(*LHARO*, *N*), cumSFE(*LHARV*, *N*) and cumSFE(*LHAROV*, *N*), respectively.

Fig 3 demonstrates the results of Table 9 from dynamic perspective. Fig 3 provide similar results with Fig 1 along with 2. In most cases, LHAROV consistently performs very closed to (even worse than) LHARV as the gray line always lay very closed to (even lower than) red line over time. The trends of gray lines are consistent with that of red lines which means the performance of LHAROV is dominated by VIX while the OVX seems redundant.

Table 10 is conducted to test whether our model can perform better than the model that only daily effects is considered. In most cases, the hypotheses that LHAROd, LHARVd, LHAROVd cannot be outperformed by LHARO, LHARV, LHAROV are rejected. Especially for *h* = 1,5, the hypotheses are rejected at high confidence level, which means the volatility forecasting performance of models that only considered the previous day information can be improved by incorporating longer lagged length.

**Table 10:** The tests of forecasting performance comparation between daily lagged and long lagged models (rolling window).

Indexes	*h* = 1			*h* = 5			*h* = 10			*h* = 22		
	HAROd	HARVd	HAROVd	HAROd	HARVd	HAROVd	HAROd	HARVd	HAROVd	HAROd	HARVd	HAROVd
SP500	0.000	0.000	0.000	0.000	0.001	0.003	0.047	0.070	0.068	0.123	0.440	0.216
Nikkei225	0.000	0.000	0.000	0.000	0.000	0.000	0.041	0.000	0.000	0.107	0.007	0.015
EUR STOXX50	0.000	0.000	0.000	0.000	0.000	0.000	0.002	0.000	0.000	0.014	0.014	0.027
FTSE100	0.000	0.000	0.000	0.000	0.000	0.000	0.013	0.011	0.029	0.025	0.091	0.077
CAC40	0.000	0.000	0.000	0.000	0.000	0.000	0.012	0.000	0.000	0.041	0.012	0.023
DAX	0.000	0.000	0.000	0.010	0.000	0.000	0.079	0.000	0.002	0.066	0.004	0.020

Notes: Conditional on Clark and West (2007) [40], values in parathesis denote the p-values of hypothesis tests with null hypothesis of LHAROd, LHARVd, LHAROVd cannot be outperformed by LHARO, LHARV, LHAROV, respectively.

### 3.5. Different model setting check

In this section, we check that whether our conclusions are robust to different model settings. Specifically, we take the average first and then take the logarithm [33] and some literatures [2, 52] take the logarithm first and then take the average. The difference between them comes from the “independent variables” rather than “dependent variables”. For instance, recall the LHARV, in our framework,

$$log{RV}_{h|t}=\beta X_{t}+\beta_{d,v}^{v}log{VIX}_{t}^{d}+\beta_{w,v}^{v}log{VIX}_{t}^{w}+\beta_{m,v}^{v}log{VIX}_{t}^{m}+\varepsilon_{t,h}$$

where $log{VIX}_{t}^{w}=log(\frac{1}{h}\sum_{i=0}^{4}{VIX}_{t-i})$; In their framework, $log{VIX}_{t}^{w}=\frac{1}{h}\sum_{i=0}^{4}{logVIX}_{t-i}$. We also consider $log{VIX}_{t}^{w}=log(\sqrt{\frac{1}{h}\sum_{i=0}^{4}{{VIX}_{t-i}}^{2}})$, the conclusions are similar.

**Fig 4:**
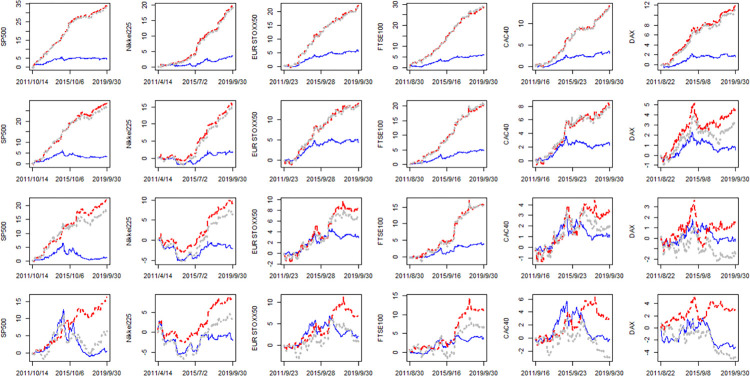
The cumSFE of LHARO, LHARV and LHAROV relative to LHAR (different model setting). Notes: The first, second, third and fourth row corresponding to *h* = 1,5,10,22, respectively. The blue line, red line and gray line denotes the cumSFE(*LHARO*, *N*), cumSFE(*LHARV*, *N*) and cumSFE(*LHAROV*, *N*), respectively.

The two model settings do not make any significant difference to our conclusions. To save space, we only present the cumSFE of LHARO, LHARV and LHAROV relative to that of LHAR (Fig 4, dynamic perspective, corresponds to Fig 1 along with 2) and corresponding tests of long lagged effects (Table 11, corresponds to Table 4 along with 8).

**Table 11:** The tests of forecasting performance comparation between daily lagged and long lagged models (different model setting).

Indexes	*h* = 1			*h* = 5			*h* = 10			*h* = 22		
	LHARO	LHARV	LHAROV	LHARO	LHARV	LHAROV	LHARO	LHARV	LHAROV	LHARO	LHARV	LHAROV
SP500	0.000	0.000	0.000	0.000	0.000	0.001	0.013	0.047	0.099	0.137	0.579	0.644
Nikkei225	0.000	0.000	0.000	0.000	0.000	0.000	0.091	0.000	0.001	0.110	0.006	0.021
EUR STOXX50	0.000	0.000	0.000	0.000	0.000	0.000	0.002	0.000	0.002	0.014	0.032	0.128
FTSE100	0.000	0.000	0.000	0.000	0.000	0.000	0.002	0.022	0.027	0.010	0.111	0.164
CAC40	0.000	0.000	0.000	0.000	0.000	0.000	0.002	0.001	0.005	0.061	0.062	0.246
DAX	0.000	0.000	0.000	0.001	0.000	0.000	0.029	0.002	0.020	0.107	0.017	0.140

Notes: Conditional on [40], values in parathesis denote the p-values of hypothesis tests with null hypothesis of LHAROd, LHARVd, LHAROVd cannot be outperformed by LHARO, LHARV, LHAROV, respectively.

Fig 4 are really similar to Fig 1 along with Fig 2. In fact, for h = 1, we find our model performs a little bit better than theirs for all indexes. But in general, there are no significant differences between these two model settings, especially for our conclusions. Moreover, Table 11 shows the hypotheses are rejected in most cases, which indicates that the longer lagged length of OVX and VIX should be incorporated into the model for volatility prediction. In a word, those model setting differences do not contaminate our conclusions.

## 4. Conclusion

This study aims to examine whether both oil price uncertainty and U.S. stock uncertainty can simultaneously provide effective information for volatility forecasting of stock market volatility. Base on HAR model, both In- and Out-of-sample empirical evidences of six major stock indexes suggest that although the information of oil market can significantly improve the performance of volatility forecasting, the improvements vanish after controlling the effects of U.S. stock uncertainty. Meanwhile, the U.S. stock uncertainty can consistently provide effective and non-negligible information for stock market volatility forecasting. Moreover, the longer lagged effects of market uncertainty spillover should be incorporated into the model as that will improve the forecasting performance.

Our study can provide important implications for the academic researchers. Previously, the literature related with oil-stock volatility spillover effects largely provide significant evidence of the existence between oil and stock volatility. In future research, the investigation of oil-stock nexus from different perspective should be considered differently. Although there exists evidence that oil market uncertainty can affects the stock market volatility, our empirical findings show the insignificant impacts of oil market uncertainty on stock market volatility after controlling the volatility spillover effects from U.S stock market. It seems puzzling, given that most literature support the significant volatility spillover from oil market to stock market, this study pave a novelty way to comprehensively understand the oil-stock nexus. Accordingly, we contribute to the existing literatures by providing empirical evidence about the insignificant impacts of oil market uncertainty on the volatility of stock market.

For market participants, this study can also provide meaningful information. The empirical findings have shown the insignificant (even negative) effects of oil market uncertainty on forecasting stock market volatility after controlling volatility spillover from U.S stock market. Therefore, given U.S stock market has been taken into account, attempts using oil market uncertainty as a key factor to predict the stock market volatility tends to deviate from desired results. Police maker should make polices to weaken the potential negative impacts from U.S market uncertainty shocks due to the strong predictive power of VIX for stock market volatility, while oil market uncertainty should not be a major factor in making stock market policies because of the insignificant incremental information provided by OVX. Investors will be benefit from the information of U.S stock market uncertainty rather than that of oil market uncertainty. They could select HAR allowing for U.S stock market uncertainty as their tools to rebalance their decisions of trading strategy and risk management and based on that, oil market uncertainty could be ignored due to its insignificant effects. Note that, due to data limitation, our conclusions are based on the six major indices we selected, the situations in other markets such as Middle east will be interesting for further investigation in future research.

## Supporting information

S1 File (CSV)
